# Expression of NgBR Is Highly Associated with Estrogen Receptor Alpha and Survivin in Breast Cancer

**DOI:** 10.1371/journal.pone.0078083

**Published:** 2013-11-04

**Authors:** Bei Wang, Baofeng Zhao, Paula North, Amanda Kong, Jian Huang, Qing Robert Miao

**Affiliations:** 1 Department of Pathology, China-Japan Friendship Hospital, Beijing, China; 2 Division of Pediatric Surgery, Department of Surgery, Children’s Research Institute, Medical College of Wisconsin, Milwaukee, Wisconsin, United States of America; 3 Divisions of Pediatric Pathology, Department of Pathology, Children’s Research Institute, Medical College of Wisconsin, Milwaukee, Wisconsin, United States of America; 4 Department of Surgery, Medical College of Wisconsin, Milwaukee, Wisconsin, United States of America; 5 Department of Pathology, Medical College of Wisconsin, Milwaukee, Wisconsin, United States of America; 6 Lester and Sue Smith Breast Center, Baylor College of Medicine, Houston, Texas, United States of America; University of L’Aquila, Italy

## Abstract

NgBR is a type I receptor with a single transmembrane domain and was identified as a specific receptor for Nogo-B. Our recent findings demonstrated that NgBR binds farnesylated Ras and recruits Ras to the plasma membrane, which is a critical step required for the activation of Ras signaling in human breast cancer cells and tumorigenesis. Here, we first use immunohistochemistry and real-time PCR approaches to examine the expression patterns of Nogo-B and NgBR in both normal and breast tumor tissues. Then, we examine the relationship between NgBR expression and molecular subtypes of breast cancer, and the roles of NgBR in estrogen-dependent survivin signaling pathway. Results showed that NgBR and Nogo-B protein were detected in both normal and breast tumor tissues. However, the expression of Nogo-B and NgBR in breast tumor tissue was much stronger than in normal breast tissue. The statistical analysis demonstrated that NgBR is highly associated with ER-positive/HER2-negative breast cancer. We also found that the expression of NgBR has a strong correlation with the expression of survivin, which is a well-known apoptosis inhibitor. The correlation between NgBR and survivin gene expression was further confirmed by real-time PCR. In vitro results also demonstrated that estradiol induces the expression of survivin in ER-positive T47D breast tumor cells but not in ER-negative MDA-MB-468 breast tumor cells. NgBR knockdown with siRNA abolishes estradiol-induced survivin expression in ER-positive T47D cells but not in ER-negative MDA-MB-468 cells. In addition, estradiol increases the expression of survivin and cell growth in ER-positive MCF-7 and T47D cells whereas knockdown of NgBR with siRNA reduces estradiol-induced survivin expression and cell growth. In summary, these results indicate that NgBR is a new molecular marker for breast cancer. The data suggest that the expression of NgBR may be essential in promoting ER-positive tumor cell proliferation via survivin induction in breast cancer.

## Introduction

Breast cancer is the most common carcinoma in women and the second most common cause of cancer death in females [1]. Early detection in conjunction with screening programs and the advent of more efficacious and targeted adjuvant systemic therapy have contributed to the decrease in breast cancer mortality [1]. The effectiveness of pathway-specific targeted and patient-tailored therapeutics demands the need for continued advances in our understanding of the molecular biology of breast cancer progression and discovery of new prognostic markers [1].

The ductal and lobular subtype constitute the majority of all breast cancers worldwide, with the ductal subtype accounting for 40–75% of all diagnosed cases [2]–[5]. Nearly 80% of all diagnosed in situ and invasive breast cancers are of ductal origin [1], [6]. In 2012, an estimated 229,060 new cases of breast cancer were expected to be diagnosed and approximately 39,920 deaths were expected to occur in the United States alone [7]. Breast cancer is the most common malignant disease in Western women, and distant metastasis are the main cause of death [6]. Here, we reveal a new potential diagnosis marker for breast invasive ductal carcinoma (**IDC**).

The Nogo isoforms-A, -B and -C are members of the reticulon family of proteins. Nogo-A and Nogo-C are highly expressed in the central nervous system (CNS), with Nogo-C also uniquely found in skeletal muscle, while Nogo-B is found in most tissues [8], [9]. Nogo-A (also called RTN4-A) binds its specific receptors, such as NgR and LiNGO1, and acts as a negative regulator of axon sprouting [10]–[13]. Nogo-B was previously identified as a protein that is highly expressed in caveolin-1 enriched microdomains of endothelial cells (**EC**) [14]. The amino terminus (residues1–200) of Nogo-B (**AmNogo-B**) serves as a chemoattractant for EC [14]. Mice deficient in Nogo-A/B show exaggerated neointimal proliferation, abnormal remodeling [14] and a deficit in ischemia induced arteriogenesis and angiogenesis [15]. NgBR was identified as a receptor specific for AmNogo-B by an expression cloning approach [16]. High affinity binding of AmNogo-B to NgBR is sufficient for AmNogo-B mediated chemotaxis and tube formation of endothelial cells [16]. We have previously demonstrated that NogoB-NgBR ligand-receptor pair is necessary for in vivo angiogenesis in zebrafish [17]. Genetic knockdown of NogoB or NgBR by antisense morpholinos abolished intersomitic vessel (**ISV**) formation during developmental angiogenesis [17]. Our recent studies further demonstrated that NgBR is essential for Ras activation in breast tumor cells [unpublished data]. However, there is no information regarding the roles of Nogo-B and NgBR in any kind of cancers, including breast cancer. Here, we demonstrate the expression patterns of Nogo-B and NgBR, their relationships with different molecular subtypes of breast cancer, and their possible roles in promoting tumor cell growth in breast cancer.

## Materials and Methods

### Tissue Microarray Slides

Three cohorts for a total of 656 breast tumor tissues and 15 normal breast tissues on tissue microarray (TMA) slides were used in this study. The first cohort, composed of 190 breast tumors and 15 normal breast tissues with duplicate cores for each case, was purchased from Shanghai Biochip Co [18]. The second cohort composed of 210 breast tumors with a single core for each case was obtained from the breast tissue bank at the Baylor College of Medicine. The third cohort composed of 256 breast tumor tissues with pathological information was purchased from BioChain (Newark, CA). All these breast cancer cases were histopathologically re-evaluated on hematoxylin and eosin-stained slides by two pathologists (BW and JH). These breast tissue specimens are anonymous and have institutional IRB (Institutional Review Board for Baylor College of Medicine) exemption.

### NgBR and Nogo-B Antibody Generation

The peptide (AHHRMRWRADGRSLEK, residues from 81–96 of NgBR) was used to immunize rabbits (Epitomics, Burlingame, CA). The antiserum was purified using the same peptide-conjugated SulfoLink Coupling Gel (Pierce, Rockford, IL). The peptide recognizing epitope 14 to 30 of human Nogo-B was used to immunize rabbits (IMG-5346A, Imgenex, San Diego, CA). In addition, NgBR rabbit monoclonal antibody (Clone ID: EPR8668) also was generated by Epitomics as a collaboration project and was used for Western blot analysis.

### Immunohistochemistry

Sections of 4 µm thickness were dried, deparaffinized and rehydrated. For all the antibodies, heat-mediated antigen retrieval was performed using steamer treatment for 20 minutes in Target Retrieval Solution (Dako S1699) before immunohistochemistry (IHC). IHC was performed using pre-diluted antibodies such as NgBR (1∶50), Nogo-B (1∶3000), survivin (1∶200), estrogen receptor alpha (**ER**, Dako, 1∶100), progesterone receptor (PR, Dako, 1∶50), Her2 (Dako, 1∶50) and CK5/6 (Dako, 1∶100). The detection system used was ImmPRESS Reagent and ImmPACT NovaRED (Vector Laboratories, Burlingame, CA). Slides were counterstained using hematoxylin. Detailed information of antibodies is shown in Table 1. To confirm the specificity of NgBR and Nogo-B IHC staining, incubation of the preabsorbed NgBR and Nogo-B antibodies using their corresponding epitope peptide-conjugated beads were considered as negative controls.

**Table 1:** Details of antibody and dilution.

Antibody	Clone	Source	Dilution
NgBR	671	Epitomics	1∶50
Nogo B	IMG-5346A	Imgenex	1∶3000
Survivin	NB500-201	Novus	1∶200
ER	SP1	Dako	1∶100
PR	PgR 636	Dako	1∶50
Her2	TAB250	Invitrogen	1∶50
CK 5/6	D5/16 B4	Dako	1∶100

Cytoplasmic and membranous staining for NgBR, Nogo-B, plasma membrane staining for Her2, nuclear reactivity for ER, PR, cytoplasmic and nuclear staining for survivin were considered positive. Quantitative scoring of NgBR, Nogo-B and survivin IHC staining was performed following previously published methods [19]. The percentage of positive cells was assigned a score from 0(0%), 1(1–10%), 2(11–25%), 3(26–50%), 4(51–75%) and 5(>75%) and the staining intensities within the respective subcellular locations were noted as 0 = negative, 1 = weak, 2 = moderate and 3 = strong. NgBR, Nogo-B and survivin staining were expressed as the score calculated by combining the staining intensity and percentage of positive cells. They were scored as negative (−, IHC score 0 to 4), weak (+, IHC score 5 to 6) and strong (++, IHC score 7 to 8). Qualitative scoring of both ER and PR was performed using ASCO/CAP criteria, i.e. 1% cell with weak staining considered as positive for ER and PR. Her2 was qualitatively/semi-quantitatively scored using ASCO/CAP guidelines, ie, Her2 scored as negative (IHC score 0 and 1+) and positive (IHC score 2+ and 3+).

#### Cell culture

MCF-7, T47D and MDA-MB-468 breast tumor cells from ATCC were grown in DMEM (Invitrogen) containing penicillin (100 U/ml), streptomycin (100 mg/ml), and 10% (v/v) fetal calf serum (HyClone) that was changed to 10% charcoal stripped FBS (GIBCO) when performing estradiol treatment.

#### siRNA transfection

NgBR siRNA1 (S1 forward: GGAAAUACAUAGACCUACA, S1 reverse: UGUAGGUCUAUGUAUUUCC), NgBR siRNA2 (S2 forward: CCAGAAUUUGCAAAUAGUA, S2 reverse: UACUAUUUGCAAAUUCUGG) oligonucleotides with 3′ dTdT overhangs were synthesized by QIAGEN (Valencia, CA). The specificity of NgBR siRNA has been validated in our previous publication [16], [17]. NgBR siRNA1 was used in all of NgBR knockdown experiments and NgBR siRNA2 was only used in experiments shown in the Figure S2. Control siRNA in experiments refers to a non-silencing siRNA (NSF: UUCUCCGAACGUGUCACGU, NSR: ACGUGACACGUUCGG AGAA) designed and synthesized by QIAGEN. MCF-7, T47D and MDA-MB-468 cells were transfected with siRNA using Oligofectamine (Invitrogen). Cell growth assay and examination of cell signaling were performed at 48–72 hours after transfection.

#### Cell growth assay

MCF-7 and T47D cells were sub-cultured to each well of 12 wells plate. After overnight culture, cells were transfected with non-silencing siRNA (NS, negative controls) or siRNA specifically targeting NgBR (siNgBR). The next day after transfection, cells were treated with 10 nM β-estradiol (Sigma). After 24 hours or/and 48 hours treatment, the total viable cell number was determined using Bio-Rad TC10™ automated cell counter Bio-Rad.

#### Western blot analysis

Total cell lysates were prepared by adding 200 µl of cell lysate buffer containing 20 mM Tris-HCl, pH 7.5, 150 mM NaCl, 1 mM EDTA, 1 mM EGTA, 2.5 mM sodium pyrophosphate, 1 mM Na3VO4, 1 mM phenylmethylsulfonyl fluoride, and 1% Triton X-100, and 1 µg/ml leupeptin. Total cell extract (50 µg) was separated on a 12% SDS-PAGE gel and transferred to a nitrocellulose membrane (Bio-Rad). Total levels of survivin, ER and NgBR were determined by using specific antibodies, survivin rabbit polyclonal antibody from Novus, ER rabbit monoclonal antibody from Dako and NgBR rabbit monoclonal antibody from Epitomics.

#### Real-time PCR

Survivin and NgBR transcripts in breast cancer were determined by real-time PCR. Normalized breast cancer cDNA arrays (BCRT101, BCRT102 and BCRT104), survivin primer and survivin standard were utilized (Origene). Total RNA was also isolated from T47D, MDA-MB-468 and MCF-7 cell lines using RNeasy mini plus kit (Qiagen). One µg RNA was used for reverse transcription (RT) using iScript cDNA synthesis kit (BioRad). The forward and reverse primers for NgBR are 5′-tgccagttagtagcccagaagcaa-3′ and 5′-tgatgtgccagggaagaaagccta-3′, respectively. The forward and reverse primers for survivin are 5′-caaggagctggaaggctg-3′ and 5′-ttcttggctctttctctgtcc-3′, respectively. Beta-actin was used as a normalized control. The forward and reverse primers for Beta-actin are 5′-ttctacaatgagctgcgtgtggct-3′ and 5′-tagcacagcctggatagcaacgta-3′ respectively. Real-time PCR analysis was performed with Bio-Rad MyiQ detection system.

### Statistical Analysis

Histological data was analyzed using statistical software SPSS 16.0 for Windows. The relationship was tested using Pearson Chi-square tests. A *p*-value<0.05 defined statistical significance. Quantitative scoring of NgBR and survivin immunostaining, real-time PCR and cell growth data are presented as mean ± the standard error of the mean (SEM) and the statistical significance of differences was evaluated with the ANOVA analysis. Significance was defined as *p*<0.05. Correlation of NgBR and Survivin were analyzed using Pearson’s correlation coefficient analysis.

## Results

### Specificity of Nogo-B and NgBR IHC Staining

To confirm the specificity of NgBR and Nogo-B IHC, we performed IHC staining in human IDC tissue sections and used primary antibodies preabsorbed with their corresponding epitope peptide-conjugated beads as negative controls. As shown in Figure S1, expression of both NgBR and Nogo-B proteins were observed only in the cytoplasm or in membrane/cytoplasm of cancer cells, and smooth muscle cells or endothelial cells of blood vessels. The expression of NgBR in smooth muscle cells was much stronger than endothelial cells. On the contrary, the expression of Nogo-B in endothelial cells was stronger than in smooth muscle cells. As negative controls, the same primary NgBR and Nogo-B antibodies were preabsorbed using their corresponding epitope peptide-conjugated beads. There are no specific staining signals in cancer cells or smooth muscle cells as well as endothelial cells of blood vessels. IHC staining using preabsorbed NgBR and Nogo-B antibodies confirmed the specificity of NgBR and Nogo-B IHC staining.

### Expression of Nogo-B and NgBR in Normal Breast Tissue

**Figure 1:**
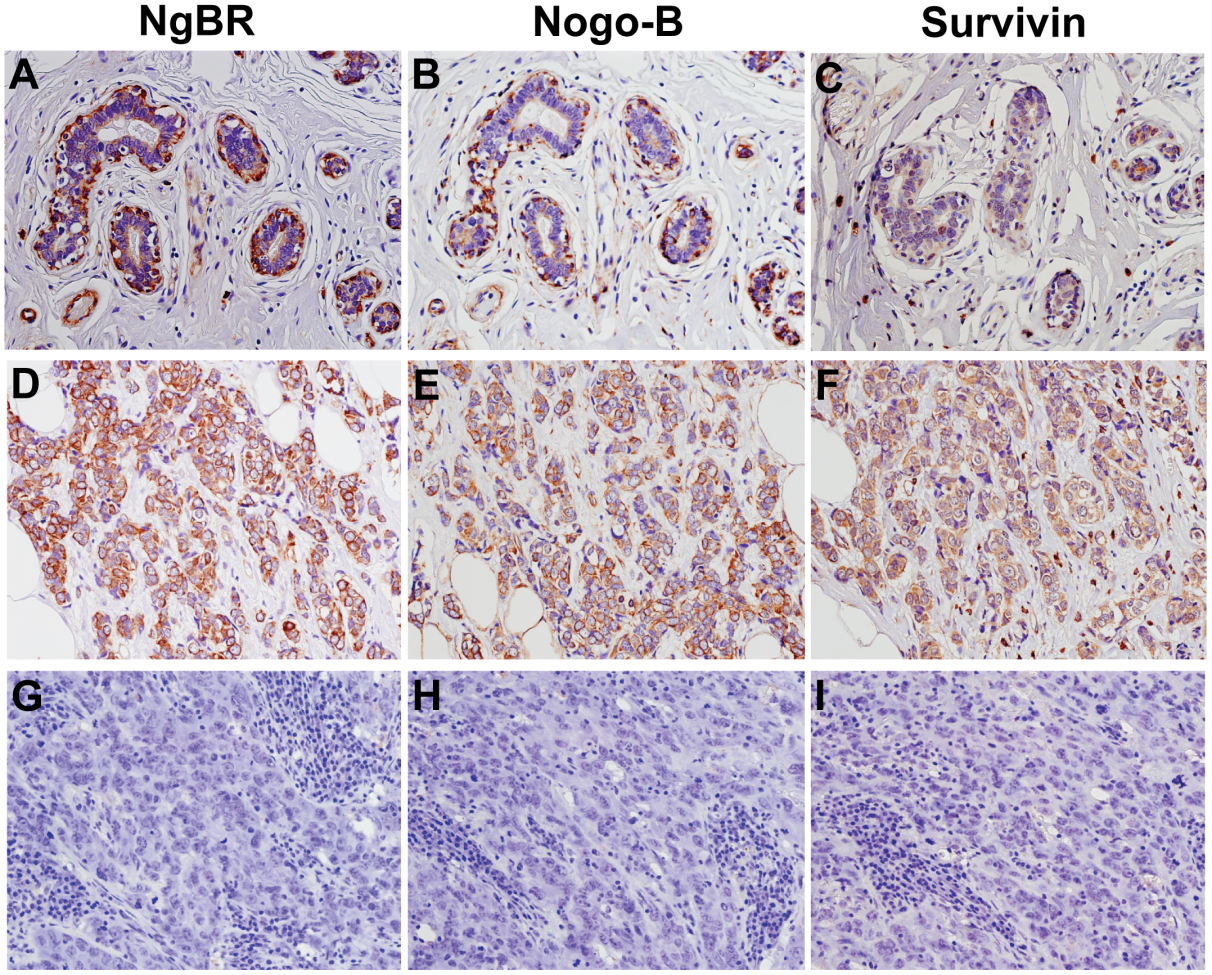
Immunohistochemical (IHC) staining of NgBR, Nogo-B and survivin in non-neoplastic breast epithelium and invasive ductal carcinoma (IDC). Staining was developed using NovaRed as described in methods. Images were taken using an Olympus microscope with x20 lens. (A–C) IHC staining of NgBR, Nogo-B and survivin in normal breast tissues. Few epithelial cells are positive for NgBR (A) and Nogo-B (B), and the majority of myoepithelial cells express NgBR (A) and Nogo-B (B). Few epithelial and myoepithelial cells are weak positive for survivin (C). (D–F) IHC staining of NgBR, Nogo-B and survivin in luminal A IDC with strongly positive staining of NgBR (D), Nogo-B (E) and survivin (F). The positive staining was localized in the cytoplasm of tumor cells with few tumor cells demonstrating membrane staining of Nogo-B and survivin. (G–I) IHC staining of NgBR, Nogo-B and survivin triple negative IDC with negative staining of NgBR (G), Nogo-B (H) and survivin (I) in all tumor cells.

As shown in Figure 1 (panel A–C), expression of NgBR, Nogo-B and survivin proteins were detected in most of the epithelial and myoepithelial cells in the normal breast tissue. The staining intensity in myoepithelial cells was stronger than in epithelial cells. In addition, the staining intensity was variable with gland-to-gland, cell-to-cell, and regional heterogeneity within a case. There is no obvious difference of expression locations between NgBR and Nogo-B in breast tissue. In addition, it is consistent with our previous discovery that expression of Nogo-B and NgBR are also detected in interstitial blood vessels of breast tissues.

### Expression of Nogo-B and NgBR in Invasive Ductal Carcinoma

Figure 1 (panel D–F) demonstrates representative IHC images of NgBR, Nogo-B and survivin in breast invasive ductal carcinoma (IDC). The overall expression of NgBR and Nogo-B in tumor cells is much stronger than in normal breast epithelial cells. The breakdown of the distribution of NgBR in breast tumors was as follows: 36.5% negative staining, 30.3% weak and 33.3% strong. Based on the scoring system previously described in the methods section, we analyzed the relationship of Nogo-B and NgBR expression with survivin expression as well as other well known breast cancer molecular subtype markers, such as ER, PR, Her2 and CK5/6. The statistical analysis results (Table 2) showed that higher expression of NgBR is frequently detected in ER-positive, and HER2-negative IDC. The expression pattern of survivin is also consistent with higher NgBR expression, namely in ER-positive, and HER-2 negative IDC. Based on molecular subtypes of IDC, NgBR is highly expressed in non-triple negative breast cancer, particularly in luminal A subtype (ER-positive and/or PR-positive, HER2-negative) of breast cancer. Although there is a strong correlation between Nogo-B and NgBR, the presence of Nogo-B as determined by IHC staining is higher than NgBR in IDC. The breakdown of the distribution of Nogo-B in IDC tumors was as follows: 8.2% negative staining, 23.5% weak and 68.2% strong. As shown in Table 2, expression of Nogo-B only has correlation with survivin, but does not have significant correlation with ER, PR, HER2 and any molecular subtypes. We further analyzed the association of NgBR and survivin expression with the progression of breast cancer. As shown in Table 3, the score of NgBR and survivin IHC staining increased in the later stages of breast cancer and the correlation of their expression in different stages of breast invasive ductal adenocarcinoma is statistically significant.

**Table 2:** Protein expression in invasive ductal carcinoma (N = 400).

Protein Level of staining N (%)	NgBR				Nogo-B				Survivin			
	−146 (36.5)	+121 (30.3)	++133 (33.3)	*p*-value	−33 (8.2)	+94(23.5)	++273 (68.2)	*p*-value	−64 (16.0)	+149 (37.2)	++187 (46.8)	*p*-value
Nogo-B												
−	29 (7.3)	3 (0.8)	1 (0.3)	0.000								
+	47 (11.8)	33 (8.3)	14 (3.5)									
++	70 (17.5)	85 (21.3)	118 (29.5)									
Survivin												
−	59 (14.8)	5 (1.3)	0 (0)	0.000	19 (4.8)	25 (6.3)	20 (5.0)	0.000				
+	59 (14.8)	66 (16.5)	24 (6.0)		11 (2.8)	37 (9.3)	101 (25.3)					
++	28 (7.0)	50 (12.5)	109 (27.3)		3 (0.8)	32 (8.0)	152 (38.0)					
ER												
negative	84 (21.0)	52 (13.0)	37 (9.3)	0.000	17 (4.3)	39 (9.8)	119 (29.8)	0.605	40 (10.0)	67 (16.8)	66 (16.5)	0.001
positive	62 (15.5)	69 (17.3)	96 (24.0)		16 (4.0)	55 (13.8)	154 (38.5)		24 (6.0)	82 (20.5)	121 (30.3)	
PR												
negative	107 (26.8)	87 (21.8)	69 (17.3)	0.000	24 (6.0)	65 (16.3)	174 (43.5)	0.430	52 (13.0)	103 (25.8)	108 (27.0)	0.002
positive	39 (9.8)	34 (8.5)	64 (16.0)		9 (2.3)	29 (7.3)	99 (24.8)		12 (3.0)	46 (11.5)	79 (19.8)	
Her2												
negative	117 (29.3)	89 (22.3)	88 (22.0)	0.031	29 (7.3)	72 (18.0)	193 (48.3)	0.079	58 (14.5)	120 (30.0)	116 (29.0)	0.000
positive	29 (7.3)	32 (8.0)	45 (11.3)		4 (1.0)	22 (5.5)	80 (20.0)		6 (1.5)	29 (7.3)	71 (17.8)	
CK5												
negative	132 (33.0)	111 (27.8)	114 (28.5)	0.257	28 (7.0)	84 (21.0)	245 (61.3)	0.692	61 (15.3)	131 (32.8)	165 (41.3)	0.231
positive	14 (3.5)	10 (2.5)	19 (4.8)		5 (1.3)	10 (2.5)	28 (7.0)		3 (0.7)	18 (4.5)	22 (5.5)	
Molecular subtype												
Triple negative	50 (12.5)	31 (7.8)	19 (4.8)	0.001	11 (2.8)	23 (5.8)	66 (16.5)	0.513	20 (5.0)	40 (10.0)	40 (10.0)	0.234
Non-triple negative	96 (24.0)	90 (22.5)	114 (28.5)		22 (5.5)	71 (17.8)	207 (51.8)		44 (11.0)	109 (27.3)	147 (36.8)	

The IHC staining levels of NgBR, Nogo-B and survivin were expressed as the score calculated by combining the staining intensity and percentage of positive cells. They were scored as negative (−, score 0 to 4), weak (+, score 5 to 6) and strong (++, score 7 to 8). N: case number; (%): percentage of total case number.

**Table 3:** Correlation analysis of NgBR and survivin in different stages of breast invasive ductal adenocarcinoma.

Stage	N (%)	NgBR Score	Survivin Score	Correlation	*p*-value
I	28 (10.9)	4.393±0.515	5.357±0.412	0.686*	0.000
II	143 (55.9)	4.734±0.213	5.615±0.178	0.730*	0.000
III	72 (28.1)	5.167±0.264	5.708±0.266	0.861*	0.000
IV	13 (5.1)	6.769±0.231	6.615±0.266	0.714*	0.015
Total	256(100)	4.912±0.149	5.669±0.128	0.746*	0.000

* *p*<0.05; N: case number; (%): percentage of total case number.

**Figure 2:**
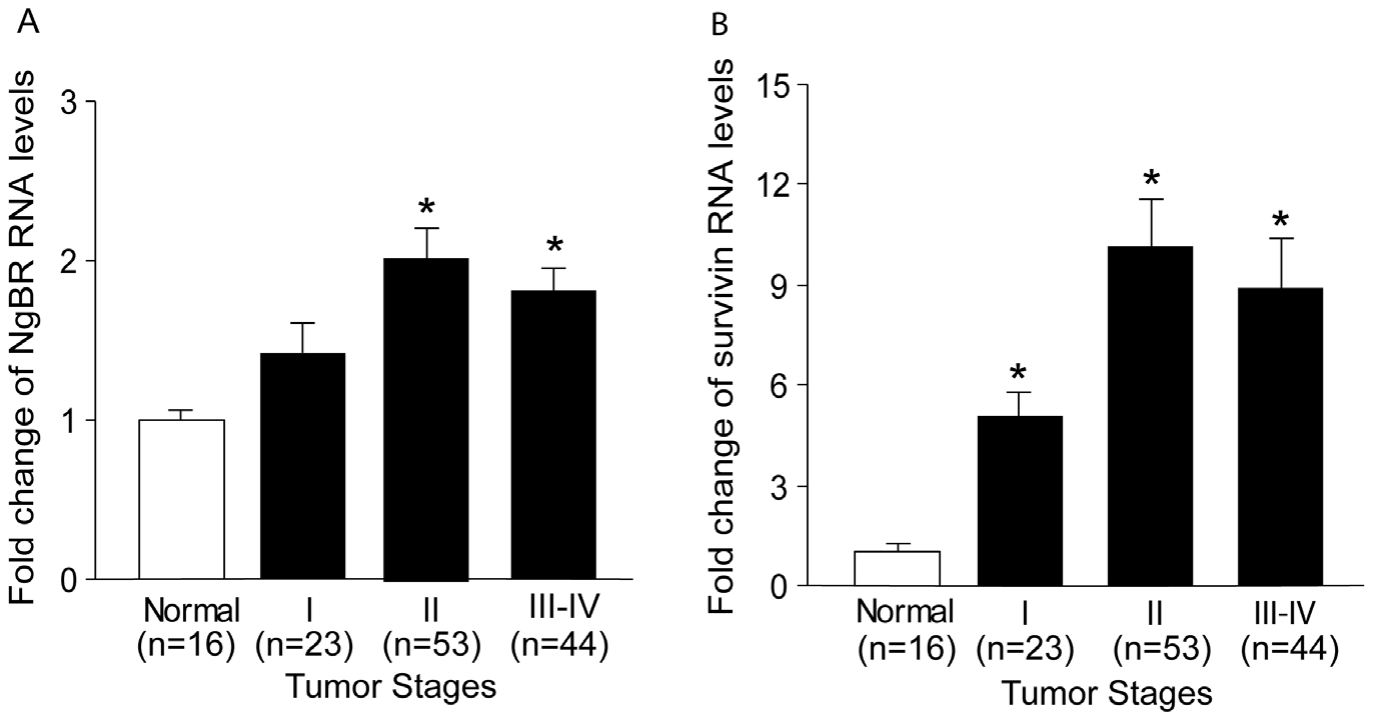
NgBR and survivin transcripts in breast tumor tissues determined by real-time PCR. Normalized human breast tumor qPCR panels were utilized (Origene). The copy number of NgBR and survivin was determined by real-time PCR. (A) NgBR RNA levels in ductal adenocarcinoma specimens were presented as fold changes as compared to the average NgBR RNA levels of all normal breast tissue (* tumor vs normal, *p*<0.05). (B) Survivin RNA levels in ductal adenocarcinoma specimens were presented as fold changes as compared to the average survivin RNA levels of all normal breast tissue (* tumor vs normal, *p*<0.05).

To further confirm the correlation of NgBR and survivin expression in breast cancer, we used a real-time PCR approach to determine the copy number of NgBR and survivin transcripts in normal and different stages of breast cancers. Three human breast tumor qPCR panels (BCRT101, BCRT102, BCRT104) were used (Origene). The panels contained a total of 136 normalized cDNAs prepared from pathologist-verified human breast tumor specimens, including 16 normal breast tissue samples, and 120 ductal adenocarcinoma tissue samples. Accompanying pathology reports were used to categorize the 120 ductal adenocarcinoma specimens into four different disease stages from I to IV. Real-time PCR results (Figure 2A) show that NgBR expression is significantly higher in Stage II (53 samples) and Stage III-IV (44 samples) ductal adenocarcinoma specimens when compared with both normal breast samples (16 samples) and Stage I ductal adenocarcinoma samples (23 samples). Consistent with NgBR expression pattern, survivin expression (Figure 2B) is significantly higher in Stage II (53 samples) and Stage III-IV (44 samples) ductal adenocarcinoma specimens when compared with normal breast samples (16 samples) and Stage I ductal adenocarcinoma samples (23 samples). We also found that expression of NgBR and survivin has statistically significant correlations in the Stage II (correlation = 0.448, *p<*0.05) and Stage III–IV (correlation = 0.386, *p<*0.05) of ductal adenocarcinoma samples, but there are no statistically significant correlations in normal and Stage I groups (Table 4). Combined with IHC staining results, our data clearly demonstrated that NgBR expression is strongly associated with survivin expression in later stages of ductal carcinomas.

**Table 4:** Correlation analysis of NgBR and survivin transcripts in different stages of ductal adenocarcinoma.

	Stage	N (%)	Correlation	*p*-value
Normal breast tissue		16(11.7)	0.077	0.776
Breast cancer stage	I	23(16.9)	0.279	0.198
	II	53(39.0)	0.448*	0.000
	III–IV	44(32.4)	0.386*	0.007
	Total	136(100)	0.432*	0.000

* *p*<0.05; N: case number; (%): percentage of total case number.

### Roles of NgBR in Regulating Survivin Expression and Cell Growth in ER-positive Breast Tumor Cells

**Figure 3:**
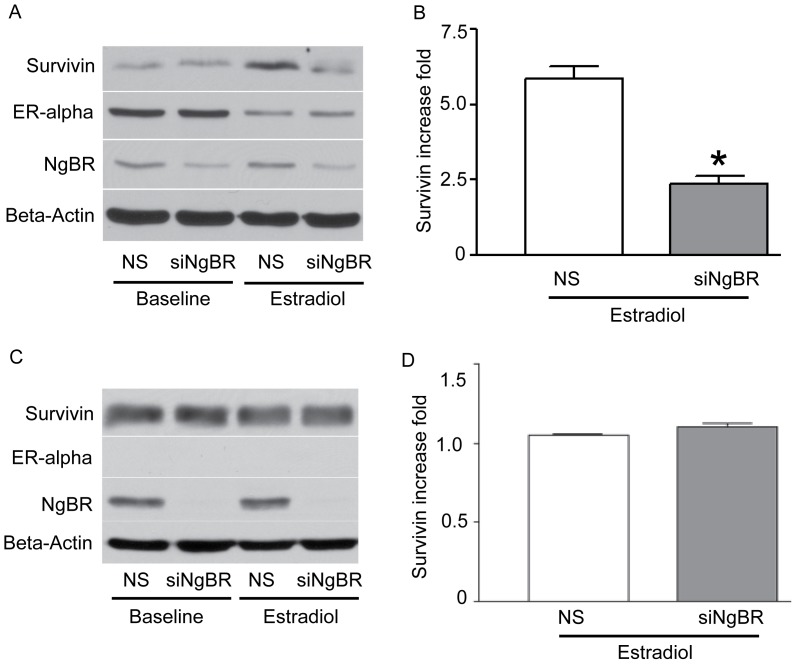
NgBR regulates estradiol-induced survivin expression in estrogen receptor positive breast tumor cells. T47D is an estrogen receptor positive breast tumor cell line. MDA-MB-468 is an estrogen receptor negative breast tumor cell line. NgBR was knockdown in both T47D and MDA-MB-468 cells using siRNA as described in methods. Both tumor cells were treated with 10 nM estradiol for 48 hours. Protein levels of NgBR, ER-alpha and survivin were determined by Western blot analysis. Beta-Actin is applied as a housekeeping protein. The density of each band was measured using NIH ImageJ and presented as relative intensity of survivin after normalized with beta-actin housekeeping protein. (A) NgBR knockdown diminished estradiol-induced survivin expression in T47D breast tumor cells. (B) Quantitative analysis of survivin protein level change in T47D cells by measuring intensity of survivin western blot band. Data is presented as fold changes of estradiol treatment group as compared to the non-treatment group (n = 3; * siNgBR vs NS *p*<0.05). (C) NgBR knockdown has no effect on survivin expression in MDA-MB-468 breast tumor cells. (D) Quantitative analysis of survivin protein level change in MDA-MB-468 cells by measuring intensity of survivin western blot band. Data is presented as fold changes of estradiol treatment group as compared to the non-treatment group. (n = 3; siNgBR vs NS *p* = 0.092).

**Figure 4:**
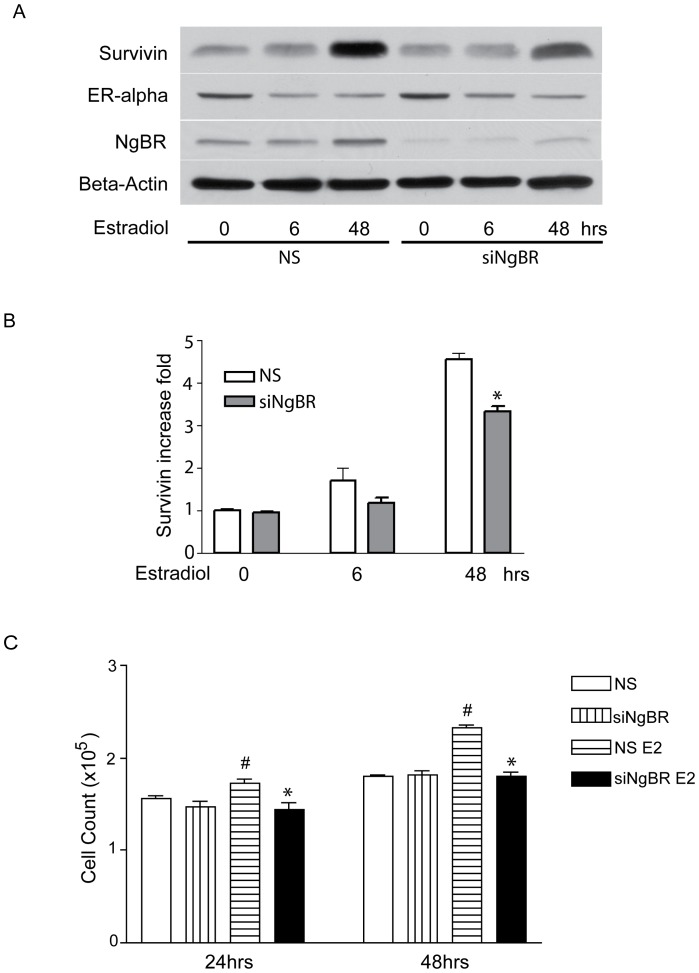
NgBR is essential for estradiol-induced survivin expression and cell growth of MCF-7 breast tumor cells. MCF-7 is an estrogen-dependent breast cancer cell line. NgBR was knocked down in MCF-7 cells using siRNA. (A) NgBR knockdown diminished estradiol-induced survivin expression in MCF-7 breast tumor cells. MCF-7 cells were treated with 10 nM estradiol for 6 and 48 hours. Protein levels of NgBR and survivin were determined by Western blot analysis. Beta-Actin is applied as a housekeeping protein. The density of each band was measured using NIH ImageJ and presented as relative intensity of survivin after normalized with beta-actin housekeeping protein. (B) Folds of survivin increase were determined by measuring relative western blot intensity of survivin. Data is presented as fold changes of estradiol treatment group as compared to the non-treatment group (n = 3; 6 hrs estradiol treatment, NS vs siNgBR *p* = 0.178; * 48 hrs estradiol treatment, NS vs siNgBR *p*<0.05). (C) NgBR knockdown impaired estradiol-stimulated cell growth of MCF-7 breast tumor cells. MCF-7 cells were treated with 10 nM estradiol for 24 and 48 hours. Viable cell numbers were counted using the Bio-Rad TC10™ Automated Cell Counter. Data is presented as mean±SEM (n = 3, # 24 hrs or 48 hrs estradiol treatment vs baseline *p*<0.05; * siNgBR vs NS *p*<0.05); E2: estradiol.

To determine the roles of NgBR in regulating survivin expression, we chose two breast tumor cell lines. One is estrogen receptor alpha positive breast tumor cell line T47D, and the other one is estrogen receptor alpha negative breast tumor cell line MDA-MB-468. We knocked down NgBR in both T47D and MDA-MB-468 cells with validated NgBR siRNA [16], [17] and examined the estradiol-induced expression of survivin. As shown in Fig. 3A and 3B, estradiol (10 nM) treatment for 48 hours increased the survivin protein levels by 5.87 fold in non-silencing (**NS**) siRNA treated T47D cells and 2.35 fold in NgBR knockdown T47D cells (NS: 5.869±0.402 vs siNgBR: 2.351±0.290, n = 3, *p<*0.05). However, as shown in Fig. 3C and 3D, estradiol (10 nM) treatment for 48hours did not significantly increase the survivin expression either in NS siRNA treated MDA-MB-468 cells or in NgBR knockdown MDA-MB-468 cells (NS: 1.054±0.008 vs siNgBR: 1.103±0.021, n = 3, *p = 0.092).* To further determine the potential roles of NgBR in regulating survivin expression and breast tumor cell growth, we knocked down NgBR in MCF-7 cells, which are a typical ER alpha positive breast carcinoma cell line, and examined the survivin expression and cell growth in response to estradiol treatment. As shown in Figure 4A and 4B, estradiol (10 nM) treatment for 6 or 48 hours increased the survivin protein levels by 1.70 or 4.18 fold in non-silencing (**NS**) siRNA treated MCF-7 cells, respectively. However, NgBR knockdown in NgBR siRNA treated MCF-7 cells reduced the estradiol-induced survivin expression (6 hr estradiol treatment, NS: 1.698±0.294 vs siNgBR: 1.184±0.114, n = 3, *p* = 0.178; 48 hr estradiol treatment, NS: 4.562±0.145 vs siNgBR: 3.319±0.134, n = 3, *p*<0.05). To confirm the specificity of NgBR siRNA, we used a second siRNA (siNgBR2) targeting the coding region of NgBR to confirm both siNgBR1 and siNgBR2 can efficiently knock down NgBR and specifically abolished the estradiol-induced expression of survivin in MCF-7 breast tumor cells (Fig. S2). In addition, we used real-time PCR approach to examine the change of survivin gene expression. As shown in the Figure S3A, estradiol treatment increases the survivin gene expression in ER-alpha positive MCF-7 and T47D breast tumor cells but not in ER-alpha negative MDA-MB-468 cells. As shown in the Figure S3B, estradiol increases the survivin gene expression in MCF-7 cells (24 hour: 1.490±0.084 fold increase) and NgBR knockdown reduces estradiol-induced survivin gene expression (24 hour: 1.021±0.096 fold increase). As shown in Fig. 3A and 4A, NgBR knockdown did not reduce estrogen receptor expression. However, estradiol treatment caused the decrease of ER-alpha levels because of ER-alpha recycling [20]_,_ [21]. In addition, we also examined the effects of NgBR knockdown on estradiol-stimulated cell growth. As shown in Figure 4C, estradiol treatment increases the growth of MCF-7 cells by 11.8% at 24 hours and 29.1% at 48 hours (n = 3, *p<*0.05), respectively, and NgBR knockdown abolishes the estradiol-stimulatory effects (24 hour: NS: 1.550±0.039×10^5^ cells vs NS+E2∶1.733±0.037×10^5^ cells, n = 3, *p<*0.05; NS+E2∶1.733±0.037×10^5^ cells vs siNgBR+E2∶1.447±0.068×10^5^ cells, n = 3, *p<*0.05; 48 hour: NS: 1.807±0.015×10^5^ cells vs NS+E2∶2.333±0.023×10^5^ cells, n = 3, *p<*0.05; NS+E2∶2.333±0.023×10^5^ cells vs siNgBR+E2∶1.800±0.0405×10^5^ cells, n = 3, *p<*0.05). Similarly, NgBR knockdown diminishes the estradiol-induced growth of T47D breast tumor cells (24 hour: NS: 7.95±0.550×10^4^ cells vs NS+E2∶12.733±0.521×10^4^ cells, n = 3, *p<*0.05; NS+E2∶12.733±0.521×10^4^ cells vs siNgBR+E2∶9.777±0.665×10^4^, n = 3, *p<*0.05). As shown in Figure 4C and Figure S4A, NgBR knockdown does not reduce the basal growth of both MCF-7 and T47D breast tumor cells, respectively. These results demonstrate that NgBR is essential for estrogen-depended survivin induction and ER positive breast tumor cell growth.

## Discussion

Although Nogo-B and NgBR have been shown to play important roles in regulating endothelial cell migration and blood vessel formation [14], [16], [17], the roles of Nogo-B and NgBR in cancer cells and cancer progression are still unclear. Nogo-B (also known as ASY) was previously identified as one of the apoptosis-inducing genes in human cancer [22]. Ectopic expression of the Nogo-B/ASY gene led to extensive apoptosis, particularly in cancer cells [22]. It was further demonstrated that Nogo-B/ASY overexpression contributes to endoplasmic reticulum stress and induces apoptosis through Ca^2+^ depletion in endoplasmic reticulum [23]. However, at the same time, stable transfectants overexpressing high levels of Nogo-B/ASY are resistant to endoplasmic reticulum stress associated stimuli, which implies that Nogo-B/ASY overexpression activates a protective response to endoplasmic reticulum stress [23]. In addition, the osteosarcoma SaOS-2 cell lines and the CHO cell lines have been shown to express high levels of endogenous Nogo-B. Overexpression of Nogo-B in both SaOS-2 and CHO cell lines do not differ significantly from the respective parental wild-type or control cell lines both in respect to cell proliferation and to spontaneous apoptosis or cell death induced by staurosporine and tunicamycin [24]. These conflicting studies have caused the uncertainty about the precise role of Nogo-B in modulating the apoptosis of cancer cells.

Our preliminary results show that overexpression of the amino-terminal domain of Nogo-B (AmNogo-B) does not cause any significant effects on tumor cell growth and cell survival (data not shown). As shown in Figure 4C and Figure S4A, knockdown of NgBR also does not affect the growth and survival of MCF-7 and T47D cells, typical estrogen receptor alpha positive breast tumor cells, under baseline growth conditions. However, NgBR knockdown reduces estradiol-induced MCF-7 and T47D cell growth (Fig. 4C and Fig. S4A), respectively. Further comparison of ER-alpha and NgBR expression in both MCF-7 and T47D cells as well as their response to estradiol stimulation show that T47D has lower ER-alpha expression and higher NgBR expression than MCF-7 cells (Fig. S4B), but T47D has more remarked response to estradiol-induced expression of survivin (Fig. S3A) as well as cell growth as compared to MCF-7 cells (Fig. S4A vs. Fig. 4C). It indicates that higher expression of NgBR may enhance the ER-alpha-mediated signaling. Our recent studies demonstrated that NgBR acts as a scaffold protein required for Ras plasma membrane translocation and Ras signaling in tumor cells [unpublished data]. Our findings suggest that NgBR may recapitulate the oncogene function of Ras and coordinate with ER to promote estrogen response. This detailed molecular mechanism needs further investigation.

Given these findings, we sought to demonstrate the significances of Nogo-B and NgBR in specific types of breast cancer. ER, PR, HER2 are three well-characterized tumor markers that are typically expressed and are strongly associated with prognosis in breast cancer. To distinguish the heterogeneity of this disease, breast cancer has been categorized as four distinct subtypes based on gene expression profiling, including luminal A (ER-positive and/or PR-positive, HER2-negative, low Ki67 index), luminal B (ER-positive and/or PR-positive, HER2-positive or HER2 negative, higher Ki67 index), HER2 enriched (HER2-positive and ER-negative/PR-negative) and triple-negative/basal like (ER-negative, PR-negative and HER2-negative) [1], [25]–[27]_,_ [28]. Luminal A and luminal B are the most common subtype, usually representing low- to intermediate-grade tumors characterized by the expression of genes that are commonly expressed by normal ductal epithelial cells [1]. The luminal A subtype is well-differentiated and associated with lobular histology and more frequent co-expression of both ER and PR than the luminal B subtype. Most cases of luminal B presented as grade II or III carcinoma showing HER2 overexpression and a higher Ki67 index [28]. The HER2 enriched subtype usually represents high-grade tumors with strong HER2 expression. The triple-negative/basal like subtype usually represents high-grade tumors displaying necrosis, prominent lymphocytic infiltration and a pushing border, carrying a poor prognosis [1], [29], [30]. Our results suggested that high expression of NgBR is positively associated with ER-positive and HER2 negative breast cancers. Our results further indicate that high expression of NgBR in ER positive breast cancer may promote tumor cell growth and division by increasing the expression of survivin via an estrogen-dependent manner. These data strongly suggest that there is a close relationship among ER alpha, NgBR, survivin and their associated signaling pathways in breast cancer. Further experiments are needed to confirm this hypothesis.

Our results clearly demonstrated that both NgBR and survivin are highly expressed in ER positive IDC (Fig. 1 and Table 2). NgBR knockdown reduced the estradiol-induced expression of survivin in ER positive breast tumor cells but not in ER-negative breast tumor cells (Fig. 3 and Fig. 4). Survivin was first identified as a baculovirus anti-apoptotic protein and is a member of the inhibitor of apoptosis proteins (IAP) family, which specifically inhibits caspases 3, 7 and 9 [31]–[33] and is involved in acquiring resistance to apoptosis. It has been shown that survivin inhibits apoptosis, regulates cell division and enhances angiogenesis [33]. Survivin is rarely expressed in terminally differentiated adult tissues, however high expression of survivin is found in most cancers [33]–[35]. High expression of survivin has been found to be related to poor survival in breast cancer patients [36], [37] and progression of breast cancer [38]. Survivin is also associated with resistance to chemotherapy and hormone therapy, and predicts a poor clinical outcome in breast cancer [37], [39]. Recent meta-analysis of survivin expression in breast cancer patients also demonstrated a significant association between positive survivin expression and a poor overall survival consequence in breast cancer patients [40]. Decreased survivin expression was found to increase sensitivity to chemotherapy drugs [41], [42] and ionizing radiation [43]. It has been shown that estrogen upregulates the expression of survivin in ER positive MCF-7 breast cancer cells [44]. This finding might suggest that there is a positive association between ER and survivin expression in breast cancer. In the context of our pathological findings in 656 specimens of breast cancer patients and in vitro results, high expression of NgBR in ER-positive breast cancer may contribute to the survivin induction caused by estrogen stimulation. Our findings further indicate that the signaling to control tumor growth may be partially mediated through the ER-NgBR-survivin pathway in ER-positive breast cancer. This pathway may serve as a potential target for directed therapy. These results suggest that NgBR may play an important role in ER-positive breast cancer growth via increasing survivin expression.

In summary, our study is the first to investigate the expression and localization of Nogo-B protein and NgBR receptor in human breast cancer. The findings from this study demonstrate: (a) NgBR is highly expressed in ER positive and Her2 negative IDC breast cancer, whereas Nogo-B is ubiquitously expressed in IDC; (b) expression of NgBR is correlated with survivin expression in IDC as well as in later stages of breast cancer; (c) NgBR is essential for estradiol-induced survivin expression in ER positive breast tumor cells; (d) and finally, NgBR is also required for estradiol-stimulated ER positive breast tumor cell growth. Although we need further investigation to reveal the molecular mechanism by which NgBR promotes survivin expression in ER-positive breast cancer cells and the potential roles of NgBR in ER-positive breast cancer progression, current findings suggest high expression of NgBR may be a novel diagnosis marker or a potential therapeutic target for ER-positive breast cancer.

## Supporting Information

Figure S1 **Immunohistochemical (IHC) staining of NgBR and Nogo-B in invasive ductal carcinoma (IDC).** Staining was developed using NovaRed as described in methods. Images were taken using an Olympus microscope with x20 lens. (A, B) To confirm the specificity of NgBR and Nogo-B IHC staining, we performed IHC staining in human IDC tissue sections and used primary antibodies preabsorbed with their corresponding epitope peptide-conjugated beads (+ peptide conjugated beads) as negative controls.
(TIF)

Figure S2 **NgBR regulates estradiol-induced survivin expression in MCF-7 cells.** NgBR was knocked down in MCF-7 cells using two different siRNA targeting NgBR (siNgBR1, and siNgBR2). Protein levels of NgBR, ER-alpha and survivin were determined by Western blot analysis. Beta-Actin is applied as a housekeeping protein.
(TIF)

Figure S3 **NgBR regulates estradiol-induced survivin gene expression in estrogen receptor positive breast tumor cells.** (A) Estradiol increases survivin gene expression in estrogen-receptor positive breast tumor cell lines (MCF-7, T47D) but not in estrogen-receptor negative cell line (MDA-MB-468). All these three cell lines were treated with 10 nM estradiol for 24 hours. Survivin gene expression was determined by real-time PCR and is normalized with beta-actin. All groups are compared to MCF-7 no estradiol treatment group. (B) NgBR regulates estradiol-induced survivin gene expression in MCF-7 cells. NgBR was knocked down in MCF-7 cells using siRNA as described in methods. The cells were treated with 10 nM estradiol for 24 hours. Survivin gene expression was determined by real-time PCR and is normalized with beta-actin. All groups are compared to NS no estradiol treatment group. E2: estradiol.
(TIF)

Figure S4 **(A) NgBR knockdown impairs estradiol-stimulated growth of T47D breast tumor cells.** Fifty thousand T47D cells were sub-cultured to each well of 12 wells plate. T47D cells were knocked down by siRNA targeting NgBR (siNgBR) and treated with 10 nM estradiol for 24 hours. Viable cell numbers were counted using the Bio-Rad TC10™ Automated Cell Counter. Data is presented as mean±SEM (n = 3, # 24 hrs estradiol treatment vs baseline *p*<0.05; * siNgBR vs NS *p*<0.05). E2: estradiol. **(B) Protein expression levels of survivin, ER-alpha and NgBR in MCF-7 and T47D breast tumor cells determined by Western blot analysis.**
(TIF)
