# Supplementary figures and images for: Expression of NgBR Is Highly Associated with Estrogen Receptor Alpha and Survivin in Breast Cancer
Source: PLoS One. 2013 Nov 4;8(11):e78083. doi: 10.1371/journal.pone.0078083 (PMC3817177; doi:10.1371/journal.pone.0078083)

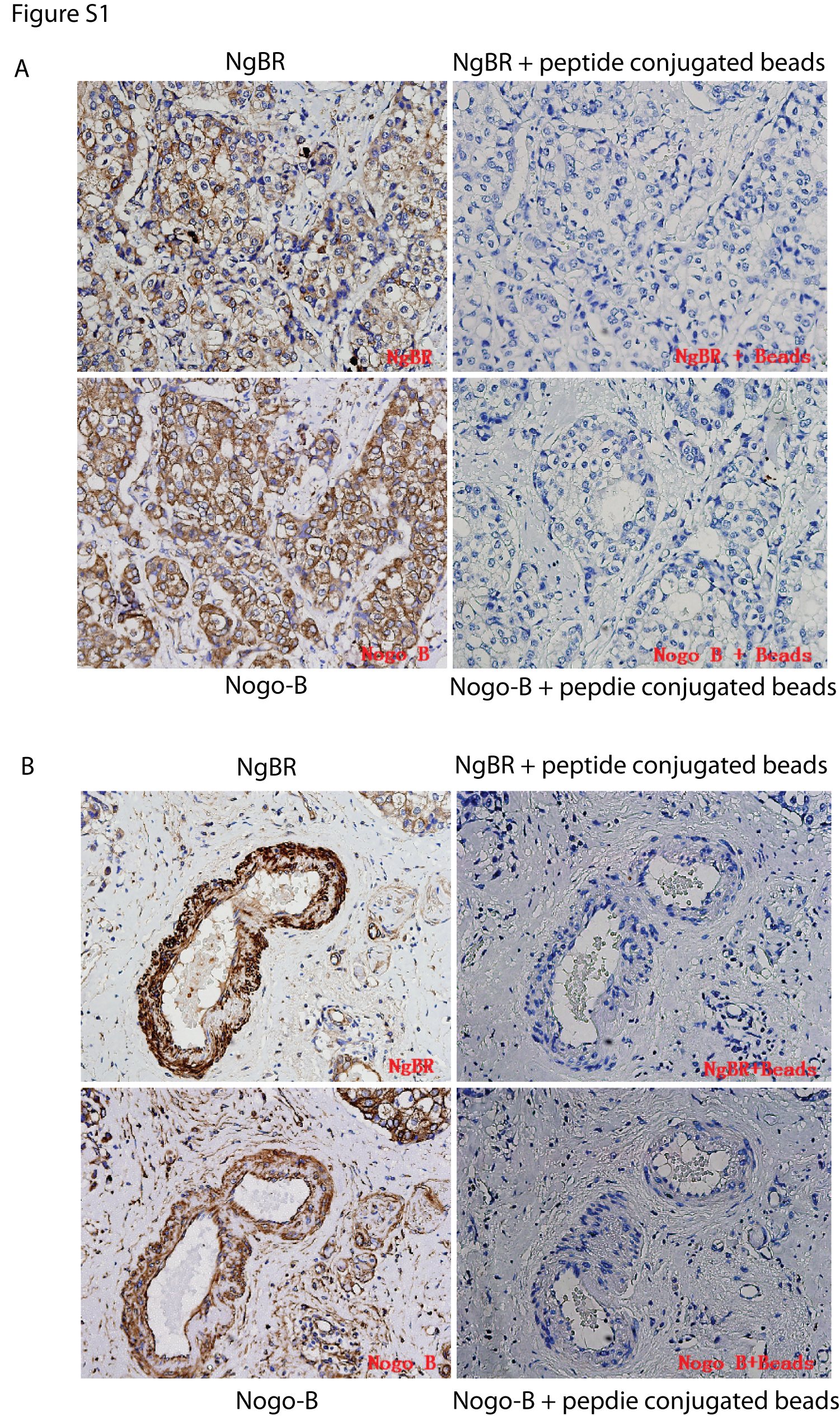

Supplement: Figure S1 — Immunohistochemical (IHC) staining of NgBR and Nogo-B in invasive ductal carcinoma (IDC). Staining was developed using NovaRed as described in methods. Images were taken using an Olympus microscope with x20 lens. (A, B) To confirm the specificity of NgBR and Nogo-B IHC staining, we performed IHC staining in human IDC tissue sections and used primary antibodies preabsorbed with their corresponding epitope peptide-conjugated beads (+ peptide conjugated beads) as negative controls. (TIF) [file pone.0078083.s001.tif]

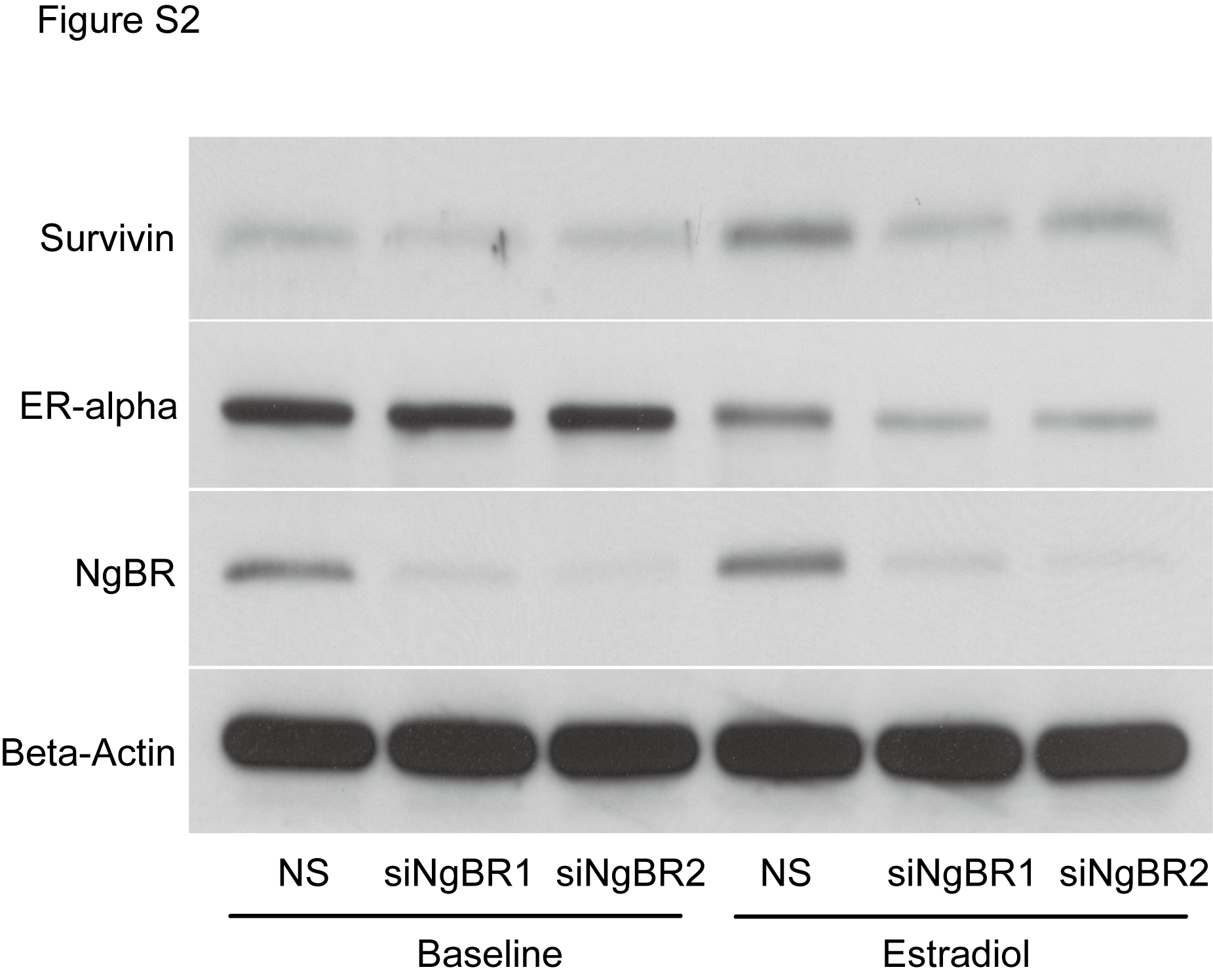

Supplement: Figure S2 — NgBR regulates estradiol-induced survivin expression in MCF-7 cells. NgBR was knocked down in MCF-7 cells using two different siRNA targeting NgBR (siNgBR1, and siNgBR2). Protein levels of NgBR, ER-alpha and survivin were determined by Western blot analysis. Beta-Actin is applied as a housekeeping protein. (TIF) [file pone.0078083.s002.tif]

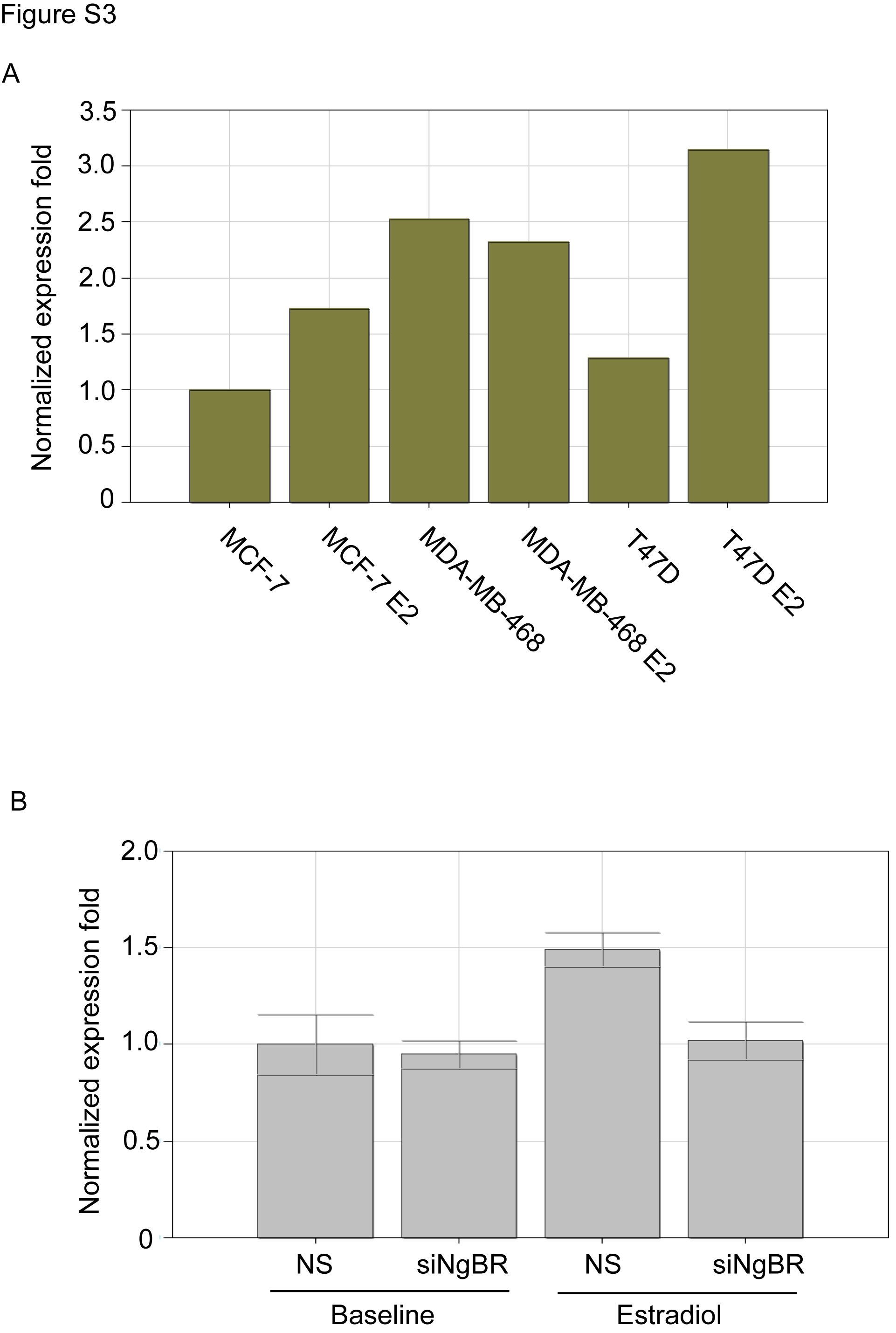

Supplement: Figure S3 — NgBR regulates estradiol-induced survivin gene expression in estrogen receptor positive breast tumor cells. (A) Estradiol increases survivin gene expression in estrogen-receptor positive breast tumor cell lines (MCF-7, T47D) but not in estrogen-receptor negative cell line (MDA-MB-468). All these three cell lines were treated with 10 nM estradiol for 24 hours. Survivin gene expression was determined by real-time PCR and is normalized with beta-actin. All groups are compared to MCF-7 no estradiol treatment group. (B) NgBR regulates estradiol-induced survivin gene expression in MCF-7 cells. NgBR was knocked down in MCF-7 cells using siRNA as described in methods. The cells were treated with 10 nM estradiol for 24 hours. Survivin gene expression was determined by real-time PCR and is normalized with beta-actin. All groups are compared to NS no estradiol treatment group. E2: estradiol. (TIF) [file pone.0078083.s003.tif]

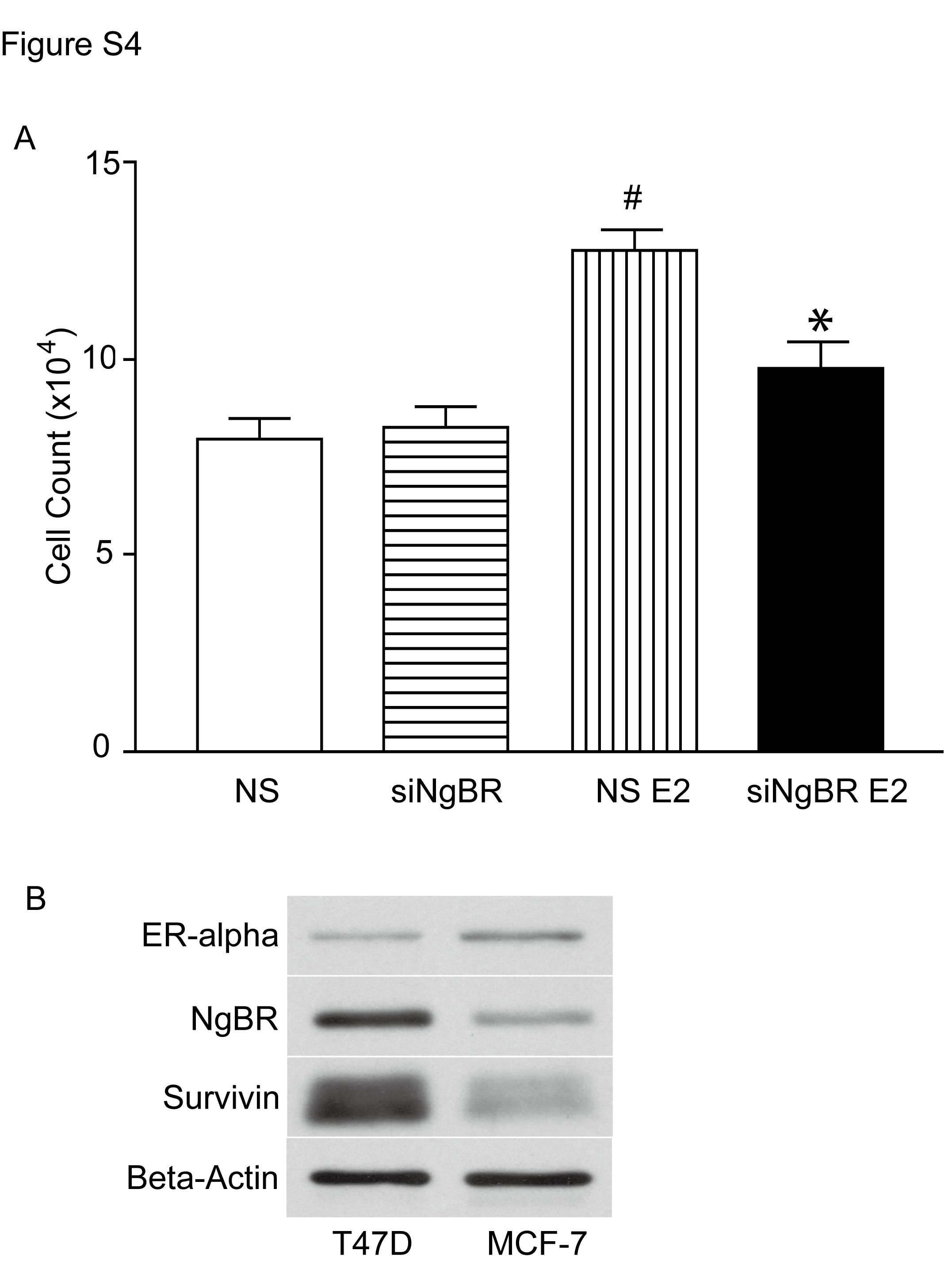

Supplement: Figure S4 — (A) NgBR knockdown impairs estradiol-stimulated growth of T47D breast tumor cells. Fifty thousand T47D cells were sub-cultured to each well of 12 wells plate. T47D cells were knocked down by siRNA targeting NgBR (siNgBR) and treated with 10 nM estradiol for 24 hours. Viable cell numbers were counted using the Bio-Rad TC10™ Automated Cell Counter. Data is presented as mean±SEM (n = 3, # 24 hrs estradiol treatment vs baseline p<0.05; * siNgBR vs NS p<0.05). E2: estradiol. (B) Protein expression levels of survivin, ER-alpha and NgBR in MCF-7 and T47D breast tumor cells determined by Western blot analysis. (TIF) [file pone.0078083.s004.tif]
